# Five years of follow-up of immediate quadriparesis caused by a large calcified mass in the ligamentum flavum: A case report

**DOI:** 10.1097/MD.0000000000017456

**Published:** 2019-10-25

**Authors:** Yafei Cao, Heng Li, Weidong Liu, Weiji Yu, Kun Gao

**Affiliations:** Department of Orthopedics and Traumatology, Shenzhen Traditional Chinese Medicine Hospital, Shenzhen, China.

**Keywords:** calcification, cervical spine, immediate quadriparesis, ligamentum flavum

## Abstract

**Introduction:**

Calcification of ligamentum flavum (CLF) is an important cause of spinal stenosis and spinal cord compression. CLF does not usually induce immediate quadriparesis. Here we describe a rare case of immediate quadriparesis due to a large calcified mass containing liquids in the ligamentum flavum, which was easily confused with gout crystals.

**Patient concerns:**

A 74-year-old Asian male felt progressive bilateral arm and leg weakness. On the fourth day, acute quadriparesis occurred.

**Diagnosis:**

Coronal and sagittal computerized tomography (CT) and magnetic resonance imaging (MRI) showed a large circular mass in the left posterior part of the cervical 3/4 spinal canal, protruding into the canal, and occupying one-half of the spinal canal.

**Interventions:**

Emergency laminectomy was performed at C3/4 level. The huge cyst was excised and 1 ml of white viscous liquid flowed out.

**Outcomes:**

After operation, CT and MRI showed a full laminectomy of C3/4 and complete decompression of the cervical spinal cord. Hematoxylin-eosin (HE) staining showed that large amounts of calcium was deposited around cystic tissues. Five-year follow-up after laminectomy showed good recovery.

**Conclusion:**

This case of immediate quadriparesis, caused by a large calcified mass containing fluid, is very rare. It should be at the earliest stage of calcification. Laminectomy is an effective treatment. This calcification was deceptive and was easily confused with gout crystals. It can help to understand the exact pathophysiology of CLF.

## 1 Introduction

Calcification of ligamentum flavum (CLF) is an important cause of spinal stenosis and spinal cord compression. As reported in several studies, it mostly occurs in Asian populations rather than in Caucasian populations.^[1]^ The exact pathophysiology is still not entirely known. Chondrocalcinosis and calcium pyrophosphate dihydrate (CPPD) crystal deposition is probably the main pathological manifestation of calcification.^[2]^

This disease occurs mostly in the thoracic and lumbar spines, but rarely in the cervical spine. The most common symptoms include numbness, dysesthesia, and pain. All these symptoms are progressive and do not suddenly worsen. Immediate quadriparesis caused by CLF is even rarer. To our knowledge, there are no reports of the relationship between CLF and immediate quadriparesis. In this report, we present the case of a patient with immediate quadriparesis caused by CPPD and 5-year follow-up after laminectomy. This case is different from other reported cases of CLF.^[3–5]^ We observed a 15 mm × 20 mm calcified cystic mass containing white fluid, which maybe the main cause of immediate quadriparesis. The liquid even confused the orthopedists and made them think it was gout crystals. This case study will help to understand the exact pathophysiology of CLF. The study was approved by the EC office of Guangzhou University of Chinese Medicine, and the rights of the subjects were protected. The patient known all rights and interests, and has signed the informed consent.

## 2 Case report

### 2.1 Clinical presentation

The patient was a 74-year-old Asian male who came to our hospital due to left limb weakness and difficulty with balance while walking, without dizziness, headache, panic or chest tightness. During hospitalization at the neurology department, he felt progressive weakness in bilateral arm and leg. No significant improvement was observed after treatment with circulatory improvement drugs. Four days later, the patient felt aggravated limb weakness and numbness and was unable to walk. The patient then had an emergency transfer from the neurology department to the orthopedics department for treatment. We conducted a motor examination, which revealed that the muscle strength of the upper extremity had markedly decreased (left biceps brachi 2/5, triceps brachii 2/5, dorsal forearm 2/5, volar forearm 2/5, hand grasp 1/5; right biceps brachi 3/5, triceps brachii 3/5, dorsal forearm 2/5, volar forearm 2/5, hand grasp 2/5). Bilateral lower extremity strength had also decreased (left quadriceps femoris 3/5, tibialis anterior 4/5, gastrocsoleus 3/5, flexor longus 1/5; right quadriceps femoris 3/5, tibialis anterior 4/5, gastrocsoleus 3/5, flexor longus 1/5). Sensation to pinprick had also decreased on the neck. Bilateral tendon hyperreflexia, positively bilateral Babinski signs and bilateral clonus hyperreflexia were also seen. The patient had a history of lacunar infarction for 6 years, and hypertension for 6 years, and had been prescribed daily use of nifedipine drugs.

### 2.2 Radiologic evaluation

**Figure 1:**
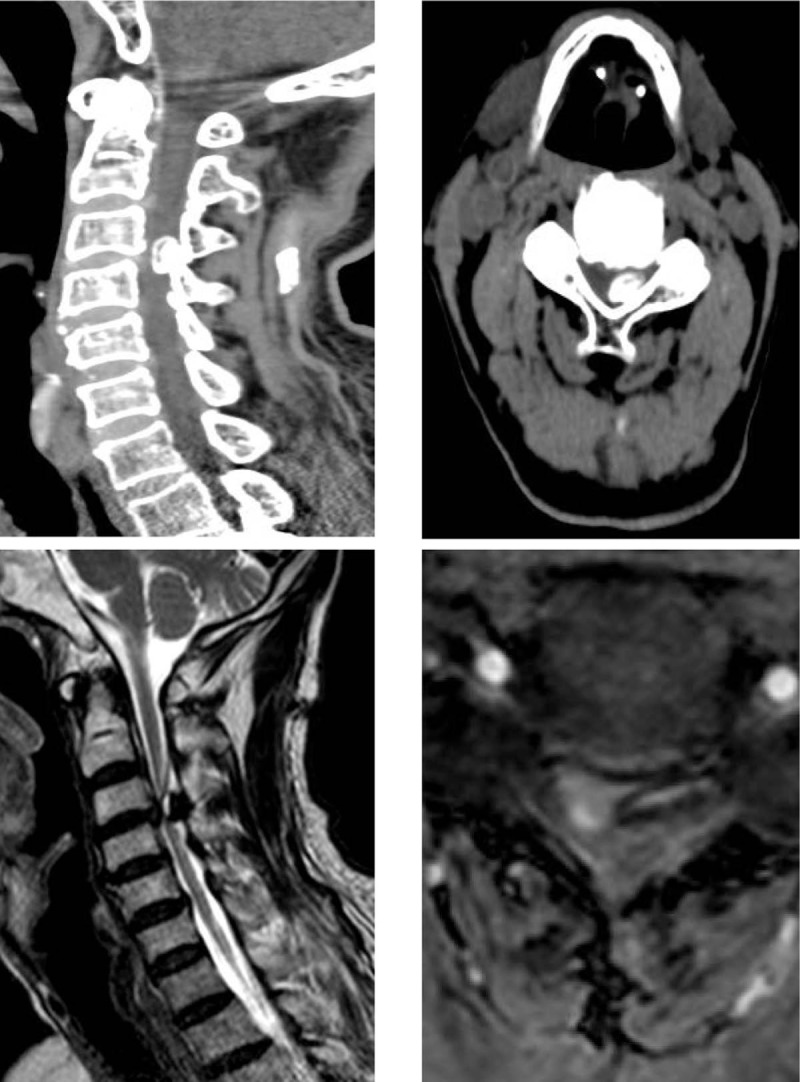
Preoperative Imaging of the cervical spine. (a, b) Axial and sagittal CT showed a circular high density mass in the left posterior part of the C3/4 spinal canal. (c, d) Axial and sagittal MRI showed a larger compression mass at C3/4 levels.

Plain radiographs showed straightening of cervical curvature, hyperosteogeny and narrowing of intervertebral spaces. Coronal and sagittal computerized tomography (CT) showed a circular high density mass in the left posterior part of the cervical 3/4 spinal canal, protruding into the canal, and occupying one-half of the spinal canal (Fig. 1a, b). Urgent magnetic resonance imaging (MRI) showed a large compression mass at C3/4 levels through high-signal intensity T1- and low-signal intensity T2-weighted MRI (Fig. 1c, d). The cervical spinal cord was compressed to thinning. No obvious mass was found in other parts of the cervical spinal canal.

### 2.3 Treatment

We urgently took the patient to the operating room, performed a laminectomy at C3/4 level with resection of the calcified mass from the ligamentum flavum. During the operation, after laminectomy, we observed a calcified ligamentum flavum tissue in the left posterior part of the dural sac, which was about 15 mm × 20 mm in size. The cyst was excised and 1 ml of white viscous liquid flowed out. The excised cyst was sent for pathological examination. Residual ligaments and calcified tissues are difficult to separate due to adhesion to the dural sac, therefore they were placed in floating treatment. Finally, the range of laminectomy was extended until the compressed dural sac was filled and restored.

### 2.4 Histopathology

**Figure 2:**
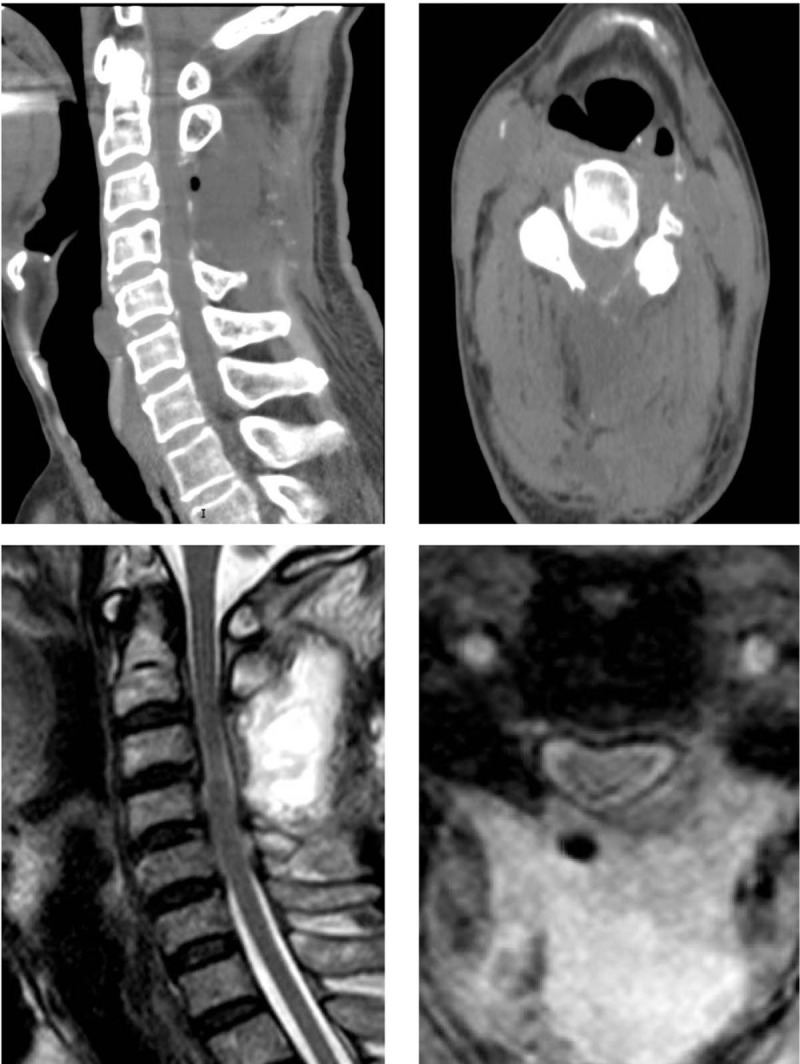
The Hematoxylin-eosin (HE) staining of the excised cyst. (a) The excised cyst. (b–d) The HE staining of excised cyst found large amounts of calcium deposits.

The excised cyst was sent for pathological examination (Fig. 2a). Hematoxylin-eosin (HE) staining showed that the specimen contained degenerated ligaments and a small amount of fibrocartilage tissue with extensive calcification. Large amounts of calcium deposits were found around the cystic tissues (Fig. 2b,c,d). Microscopically, no gout crystals were found in the liquid. However, unfortunately, we were unable to collect the white liquid in time during the operation. We were only able to see through the naked eye that it was a white, sticky liquid.

### 2.5 Outcome and follow-up information

**Figure 3:**
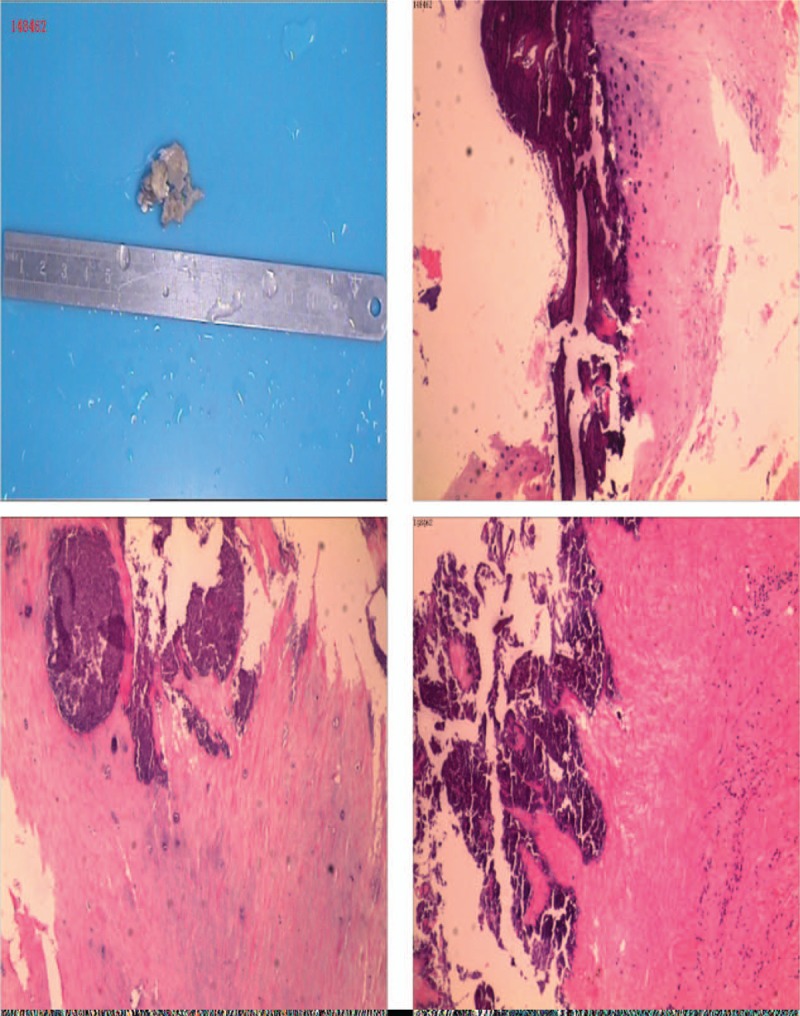
Postoperative Imaging of the cervical spine. (a, b) Axial and sagittal CT showed a full laminectomy of C3/4. (c, d) Axial and sagittal MRI showed complete decompression of the cervical spinal cord.

**Figure 4:**
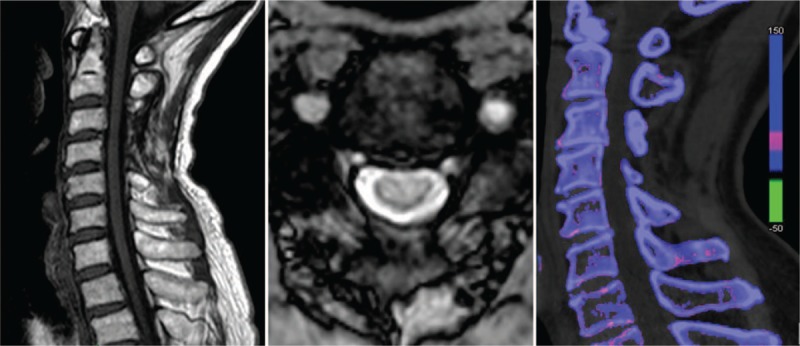
Fifth year follow-up imaging. (a, b) Axial and sagittal MRI showed complete decompression of the cervical spinal cord. (c) Dual-energy CT showed no gout deposits appeared in the cervical spine, as green represents gout.

After surgery, CT showed a full laminectomy of C3/4 (Fig. 3a, b), and MRI showed complete decompression of the cervical spinal cord (Fig. 3c, d). On the second day after operation, subcervical sensation of the patient had recovered significantly. Two weeks later, the patient was able to walk, lift upper limbs, and touch his head. Six months later, muscle strength of the limbs had returned to normal, and 1 year later, he could jog. Three years later, he was able to live a normal life. Approximately 5 years after the operation, he had mild complaints regarding his neck and left shoulder. Muscle strength of extremities was normal. MRI showed that the spinal cord shape was normal, without compression (Fig. 4a, b). Dual-energy CT showed that no gout deposits appeared in the cervical spine (Fig. 4c).

## 3 Discussion

There are several special features of this case. The patient suffered from acute quadriparesis. Without timely decompression, quadriparesis would have been irreversible and even life-threatening. In addition, during the operation, we observed a 15 mm × 20 mm calcified cystic mass containing 1 ml of a white viscous liquid. This has never been reported before. Immediate quadriparesis was most likely caused by a sharp increase of this liquid.

Spinal disease through CPPD crystal deposition has been repeatedly reported.^[5–7]^ CLF is the most common issue. Nanko reported of a 70-year-old woman with radiculomyelopathy at C5–6 and C6/7 level in 1976, bringing attention to the fact that CLF is a cause of disease for the first time.^[8]^ In 1983, Yoshinobu reported of 4 cases of calcification of the cervical ligamentum, and noticed that calcification might be induced by the degeneration or abnormal nutritional state of the ligamentum flavum.^[4]^ Wada reported of a 54 year old male with neck pain, who was preoperatively diagnosed with CLF.^[9]^ Intraoperatively it was found to be CPPD crystal deposition along the spinal dura mater. Various factors, including the aging process, chondrocytic metaplasia, endocrine imbalance, metabolic diseases, and mechanical stress, have been reported as major causes of the development of CLF.^[10,11]^ Pathological change studies speculated that during the early stages, CPPD deposited in the ligamentum flavum, and then during the middle and late stages, CPPD is transferred to a stable final form of hydroxyapatite crystals.^[2,11]^ In our case, immediate quadriparesis was caused by a large calcified mass, and the 15 mm × 20 mm calcified cystic mass contained 1 ml of a white viscous liquid. This white viscous liquid may be the earliest form of CPPD aggregation that had not yet been calcified.

In this case study, the rapidly progressive course and liquid released during surgery confused the orthopedists, and made them think it was gout crystals. However, histopathology did not support this suggestion. The uric acid level of the patient was normal and no arthritis was detected in other joints. Prior to surgery, it is difficult to distinguish calcification and gout. Recently, along with the development of dual-energy imaging technology, a new option of imaging examination of gout has been made available. Its work principle is that different energy CT values of human tissues can be processed by software to display characteristic color coding for the chemical composition of tissues. Dual-energy CT can show the location and quantity of gout deposits.^[2,5,12]^ But the gold standard for diagnosis of spinal gout is still monosodium urate crystallization pathology. Jegapragasant declared that for patients with lower back pain or nerve compression symptoms, spinal gout should be highly suspected if the patient has a history of gout, especially multiple nodules, hyperuricemia, and characteristic imaging changes.^[5]^ As reported by Takashi, a 70-year-old Japanese woman, was affected by pseudogout of the cervical yellow ligament, leading to neck pain. C6 laminectomy and removal of the C5-C6 yellow ligament was able to achieve a satisfactory therapeutic effect.^[6]^ Cervical laminectomy is likely to result in kyphosis deformity.^[13]^ However, according to the 5 year observation, the cervical curvature of the patient had recovered well.

In conclusion, this study demonstrates a case of immediate quadriparesis caused due the presence of a large calcified mass in the ligamentum flavum. The 5-year minimum follow-up period showed good results, proving that laminectomy is an effective treatment. This calcification was deceptive and was easily confused with gout crystals. This case study will help us to understand the exact pathophysiology of CLF.

## Author contributions

**Conceptualization:** Weiji Yu, Yafei Cao.

**Data curation:** Kun Gao, Heng Li, Weidong Liu, Weiji Yu.

**Formal analysis:** Kun Gao, Heng Li.

**Funding acquisition:** Yafei Cao.

**Investigation:** Kun Gao, Heng Li.

**Supervision:** Weidong Liu, Weiji Yu.

**Writing – original draft:** Kun Gao.

**Writing – review & editing:** Kun Gao.

Yafei Cao orcid: 0000-0001-9193-376X.

## References

[1] Ahn DK Lee S Moon SH Ossification of the Ligamentum Flavum. Asia Spine J 2014;8:89 96. 10.4184/asj.2014.8.1.89 PMC3939377 24596612

[2] Brown TR Quinn SF D’Agostino AN Deposition of calcium pyrophosphate dihydrate crystals in the ligamentum flavum: evaluation with MR imaging and CT. Radiology 1991;178:871 3. 1994435 10.1148/radiology.178.3.1994435

[3] Amato V Giannachi L Irace C Thoracic spinal stenosis and myelopathy: report of two rare cases and review of the literature. J Neurosurg Sci 2012;56:373 8. 23111299

[4] Iwasaki Y Akino M Abe H Calcification of the ligamentum flavum of the cervical spine. Report of four cases. J Neurosurg 1983;59:531 4. 6886767 10.3171/jns.1983.59.3.0531

[5] Jegapragasan M Calniquer A Hwang W A case of tophaceous gout in the lumbar spine: a review of the literature and treatment recommendations. Evid-Based Spine-Care J 2014;5:52 6. 24715872 10.1055/s-0034-1366979 PMC3969431

[6] Kobayashi T Miyakoshi N Abe T Acute neck pain caused by pseudogout attack of calcified cervical yellow ligament: a case report. J Med Case Rep 2016;10:1 4. 27237823 10.1186/s13256-016-0928-1 PMC4885118

[7] Zhu D Chen Y Lumbar idiopathic intervertebral disc calcification associated with ossification of the ligamentum flavum in adult: a case report. Br J Neurosurg 2018;32:579 81. 28703022 10.1080/02688697.2017.1354120

[8] Nanko S Takagi A Mannen T A case of cervical radiculo-myelopathy due to calcification of the ligamentum flavum. Neurol Med 1976;4:205 10. [in Japanese].

[9] Wada N Yamashita K Hiwatashi A Calcium pyrophosphate dihydrate crystal deposition disease of the spinal dura mater: a case report. BJR Case Rep 2017;4:20170049. 30363166 10.1259/bjrcr.20170049 PMC6159146

[10] Sato K Hayashi M Kubota T Symptomatic calcification and ossification of the cervical ligamentum flavum: clinical, radiological and pathological features. Br J Neurosurg 1989;3:597 602. 2818852 10.3109/02688698909002852

[11] Takahashi T Hanakita J Minami M Pathophysiology of calcification and ossification of the Ligamentum flavum in the cervical spine. Neurosurg Clin N Am 2018;29:47 54. 29173435 10.1016/j.nec.2017.09.016

[12] Kaler J Mukhtar O Khalid M Spinal gout causing reversible quadriparesis: a case report and literature review. J Commun Hosp Internal Med Perspect 2018;8:111 4. 10.1080/20009666.2018.1472515 PMC5998288 29915646

[13] Morino T Ogata T Horiuchi H Eight years of follow-up after laminectomy of calcium pyrophosphate crystal deposition in the cervical yellow ligament of patient with Coffin–Lowry syndrome. Medicine 2016;95:e4468. 27495083 10.1097/MD.0000000000004468 PMC4979837

